# Multi-Scale Convolutional Neural Network for Accurate Corneal Segmentation in Early Detection of Fungal Keratitis

**DOI:** 10.3390/jof7100850

**Published:** 2021-10-11

**Authors:** Veena Mayya, Sowmya Kamath Shevgoor, Uma Kulkarni, Manali Hazarika, Prabal Datta Barua, U. Rajendra Acharya

**Affiliations:** 1 Healthcare Analytics and Language Engineering (HALE) Lab, Department of Information Technology, National Institute of Technology Karnataka, Surathkal, Mangalore 575025, India; sowmyakamath@nitk.edu.in; 2 Department of Information & Communication Technology, Manipal Institute of Technology, Manipal Academy of Higher Education (MAHE), Manipal 576104, India; 3 Department of Ophthalmology, Yenepoya Medical College, Yenepoya (Deemed to Be University), Mangalore 575018, India; UmaKulkarni@yenepoya.edu.in; 4 Cornea and Anterior Segment Services, Department of Ophthalmology, Kasturba Medical College, Manipal Academy of Higher Education (MAHE), Manipal 576104, India; 5 School of Management & Enterprise, University of Southern Queensland, Toowoomba, QLD 4350, Australia; Prabal.Barua@usq.edu.au; 6 Faculty of Engineering and Information Technology, University of Technology, Sydney, NSW 2007, Australia; 7 School of Engineering, Ngee Ann Polytechnic, Clementi 599489, Singapore; Rajendra_Udyavara_ACHARYA@np.edu.sg; 8 Department of Biomedical Engineering, School of Science and Technology, Singapore University of Social Sciences, Singapore S599494, Singapore; 9 Department of Biomedical Informatics and Medical Engineering, Asia University, Taichung 41354, Taiwan

**Keywords:** clinical decision support systems, fungal keratitis, microbial keratitis, cornea segmentation, slit-lamp images, convolution neural networks

## Abstract

Microbial keratitis is an infection of the cornea of the eye that is commonly caused by prolonged contact lens wear, corneal trauma, pre-existing systemic disorders and other ocular surface disorders. It can result in severe visual impairment if improperly managed. According to the latest World Vision Report, at least 4.2 million people worldwide suffer from corneal opacities caused by infectious agents such as fungi, bacteria, protozoa and viruses. In patients with fungal keratitis (FK), often overt symptoms are not evident, until an advanced stage. Furthermore, it has been reported that clear discrimination between bacterial keratitis and FK is a challenging process even for trained corneal experts and is often misdiagnosed in more than 30% of the cases. However, if diagnosed early, vision impairment can be prevented through early cost-effective interventions. In this work, we propose a multi-scale convolutional neural network (MS-CNN) for accurate segmentation of the corneal region to enable early FK diagnosis. The proposed approach consists of a deep neural pipeline for corneal region segmentation followed by a ResNeXt model to differentiate between FK and non-FK classes. The model trained on the segmented images in the region of interest, achieved a diagnostic accuracy of 88.96%. The features learnt by the model emphasize that it can correctly identify dominant corneal lesions for detecting FK.

## 1. Introduction

The cornea is a transparent layer of tissue covering the front surface of the eye that acts as a window, allowing light to enter the eye. Infection is the most common cause of corneal ulcers (keratitis), and at least 4.2 million people worldwide are reported to suffer from corneal opacities, according to the 2019 World Vision Report [1]. Corneal opacity is caused by a variety of conditions that cause the cornea to scar or become opaque. Microbial keratitis (MK) or infectious keratitis (IK) is the primary cause of corneal opacification, and the fifth leading cause of visual impairment in the developing world [2]. If such infections are not detected and treated early, they can cause irreversible corneal blindness due to perforation, endophthalmitis and panophthalmitis [3,4,5,6]. FK in particular, is challenging to treat at later stages and may necessitate surgery.

The gold standard method for diagnosing FK is corneal scraping with microbiological culture-sensitivity testing. However, this is a time-consuming laboratory procedure [7]. Fungal organisms are slow growing and may not be florid, in the early stages of FK. Fungal cultures may also have limited sensitivity due to the scant quantity of material accessible from corneal scrapings, which may in turn lead to false-negative results [7]. Slit-lamp examination of the ocular surface, particularly the cornea, conjunctiva, and anterior chamber, is widely used in the diagnosis of MK. However, the findings of corneal staining combined with slit-lamp biomicroscopy is heavily reliant on the grader’s clinical knowledge. It has been reported that correctly differentiating between bacterial keratitis and FK is a challenging process even for trained corneal experts and is often misdiagnosed in more than 30% of the cases [8]. Furthermore, certain clinical signs typically attributed to FK may also be of bacterial or protozoal origin, thereby complicating the diagnostic process. Automated grading of FK images could overcome these limitations by lowering physician burden and improving patient prognosis through early diagnosis.

Over the years, there has been significant interest in leveraging the predictive power of Artificial Intelligence (AI) based models to facilitate infectious ocular disease diagnosis [9,10] and measure its severity through lesional segmentation [11,12]. For clinical decision support systems (CDSS) to be adaptable in real-world circumstances, providing a transparent, explainable decision (even if it is wrong) is considerably more acceptable than putting forth a highly accurate, non-transparent decision. In this work, we propose an intelligent diagnostic system for early detection of FK using slit-lamp images, supporting the established diagnosis. A multi-scale convolutional neural network (MS-CNN) is proposed to segment the corneal region for in-depth visual examination. Deep neural models are employed for the early diagnosis of FK, and features learnt by the model for prediction are visualized to aid model explainability and evidence-based diagnosis.

The rest of the paper is organized as follows: Section 2 presents a detailed overview of existing techniques employed for FK diagnosis. Section 3 documents the proposed methodology for data collation, corneal region segmentation, classification and lesion visualization. Section 4 details the experiments conducted to evaluate the performance of the proposed approach and the benchmarking performed for comparative evaluation against state-of-the-art works. The merits and limitations of the proposed technique are discussed in Section 5. Section 6 summarizes the proposed experimental study and presents the future work.

## 2. Background

The primary causative factors for FK are prolonged contact lens wear, topical steroid usage, trauma caused due to organic or vegetative matter, pre-existing systemic disorders, and other ocular surface issues [13]. It is a serious public health problem resulting in significant morbidity, if left untreated. Early interventions and treatment are critical for the recovery and prevention of corneal blindness [13,14]. Although ophthalmologists are trained to diagnose FK based on specific clinical signs and symptoms, isolation of fungi in micro-biological culture-based techniques remains the gold standard for diagnosis. However, these are time-consuming and labor-intensive [15]. The most clinical signs based on which ophthalmologists differentiate corneal ulcers are infiltrate location, pattern, depth, epithelial defect size, surrounding stromal haze, and the presence (or absence) of hypopyon. Specifically, FK is associated with the occurrence of an uneven or feathery border, raised profile, deep stromal infiltrates, satellite lesions, endothelial plaques and/or pigmentation [16,17]. As per studies conducted, general ophthalmologists typically differentiate FK from non-FK about 49.3–67.1% of the time, while trained corneal specialists can distinguish FK from non-FK 66.00–75.90% of the time [8,16,18]. These statistics are a significant cause for concern and highlight the need for effective automated diagnostic systems to assist doctors in the early detection of FK. Automated systems can aid in the timely diagnosis of FK by means of tele-ophthalmology in rural areas where there is a shortage of doctors.

AI based CDSS have achieved promising performance in identifying a variety of eye disorders [19,20,21]. However, very few studies have used AI to enable the early diagnosis of FK using digital slit-lamp images. Loo et al. [12] proposed a modified version of mask R-CNN (region-based CNN) called SLIT-Net for the segmentation of ocular structures and biomarkers using 133 MK digital slit-lamp images. However, the authors did not address the problem of classification of keratitis based on microbial etiology. Xu et al. [9] developed a patch-level deep model to classify IK by manually segmenting the infectious regions of slit lamp microscopy images. The manual segmentation of corneal areas is a time-consuming task and needs expertise. Kuo et al. [10] collected a total of 288 microbial laboratory-confirmed images, which included 114 FK and 174 non-FK slit-lamp biomicroscopy images. The authors used DenseNet [22] for their experiments and performed five fold cross-validation to classify between FK and non-FK. They reported an average accuracy of about 70% and also highlighted that incorporation of transfer learning and RoI cropping processes could contribute to further improvements in the performance. Furthermore, RoI segmentation results in uniform clarity in images, which also helps enhance performance while also enabling accurate evidence for trustworthy prediction. Recently, Hung et al. [23] used U${}^{2}$ Net [24] to segment the cornea in slit-lamp biomicroscope images and then incorporated transfer learning to classify these images into bacterial and FK. The authors directly utilized the U${}^{2}$ Net segmented corneal regions and achieved an average diagnostic accuracy of 80%. The segmented masks contained noisy boundaries, clipped cornea regions, and other features that could have erroneously contributed to predictions, as evidenced from the heat map generated using the corneal segmented images.

Based on the extensive literature review, it can be observed that there is ample scope for building completely automated diagnostic systems, with high sensitivity and specificity, in addition to enabling evidentiary support for predictions. Our study addresses several lacunae in existing approaches, by utilizing automated corneal region segmentation to enhance the performance of evidence-based CDSS. In contrast to existing works, we propose a two-stage approach that can potentially improve the performance of the etiological classification while also providing reliable evidence for predictions. In the next section, we present a detailed discussion on the proposed methodology adopted for early detection of FK.

## 3. Materials and Methods

**Figure 1:**
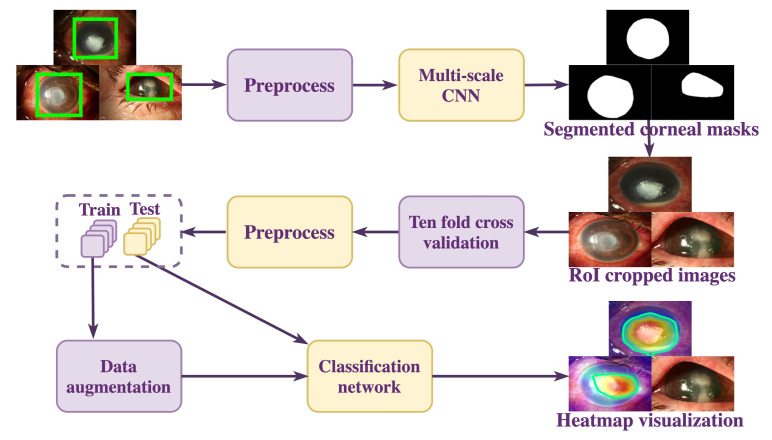
Proposed methodology for FK classification.

The overall workflow of the proposed approach is depicted in Figure 1. The slit-lamp biomicroscopy images tend to contain noise in the form of surrounding background that may affect the classification model’s prediction. To enable the classification model to learn the minute changes in the corneal region, we segmented the RoI using the proposed MS-CNN, after which the cropped RoI images are preprocessed and classified using a classification network. To highlight the features of an input slit-lamp image that the classification model considered relevant for prediction, the features were visualized using Gradient-weighted Class Activation Mapping (Grad-CAM) [25]. Each process depicted is described in more detail in subsequent sections.

### 3.1. Data Collation

For the experimental evaluation of the proposed approach, Loo et al. [12]’s dataset consisting of 133 clinically suspected MK images was used. We also collated around 540 public-domain images containing both FK (250) and non-FK images (290). Of the 290 images, 150 images were of *viral* keratitis, 120 images of *bacterial* keratitis and 20 images of *acanthamoeba* keratitis. These were obtained from various Web sources, and all are microbiologically validated cases which were used for meta-analysis purposes. This study was exempted from the purview of ethical clearance by Yenepoya Ethics Committee-1 (YEC-1/2021/046). The dataset provided by Loo et al. [12] was analysed by two ophthalmologists and labelled as *fungal*(1) or *non-fungal*(0), based on clinical observations. The clinically suspected MK images were assigned to the FK group if at least one of the ophthalmologists who participated in the study identified it as FK. Similarly, when both ophthalmologists labelled the images with non-FK, the images were assigned to the non-FK group. The corneal region annotation was performed using VGG Image annotator [26], after which the mask images were formed using the annotated regions.

### 3.2. Data Preprocessing and Augmentation

The collated data was preprocessed and augmented before the RoI segmentation and classification phases. We used the CLAHE algorithm (Contrast Limited Adaptive Histogram Equalization) [27] algorithm to increase the contrast and highlight the corneal border. All the images and the corneal masks were resized to 512×512. Before categorising the images into FK and non-FK, the images were scaled to (width=384×height=256) based on the normal distribution of the training images. The images were augmented by vertical and horizontal flipping of images to prevent the overfitting of the model. Rotated images at random angles ranging from 200${}^{{}^{\circ}}$ to 360${}^{{}^{\circ}}$ degrees were also included in each training batch.

### 3.3. Multi-Scale CNN Model for RoI Segmentation

**Figure 2:**
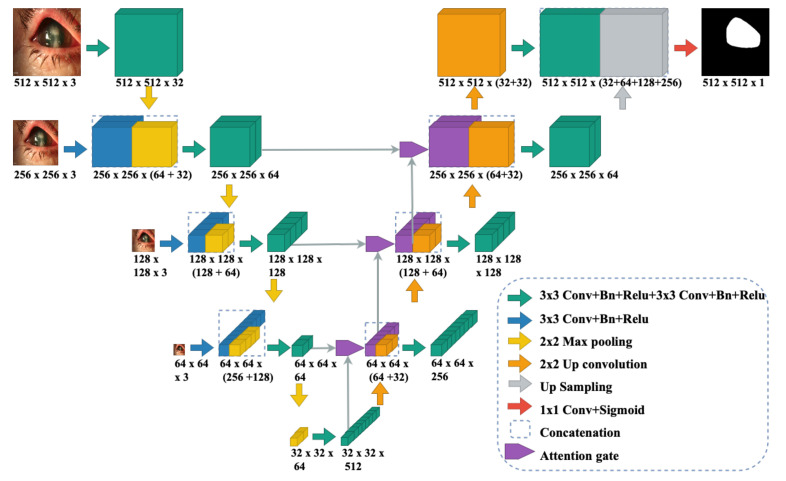
MS-CNN architecture used for corneal RoI segmentation.

Since the data collated in this study included images of varying dimensions, a MS-CNN model is proposed for accurate segmentation of the corneal region. The network architecture is shown in Figure 2 and is based on UNet [28] and attention UNet [29] architectures, which work well with modest training data. After improving the contrast of the corneal boundaries using CLAHE, the images were passed through a succession of convolution, and max-pooling layers for local feature extraction. The expansion layers were utilised to re-sample the image maps using extracted contextual information. Skip connections were utilised to encourage more semantically relevant outputs and handle varying resolution images to mix high-dimensional local characteristics with low-dimensional global information. The output of each dimension is then up-sampled and concatenated with the output from the first dimension. Ultimately, the resultant concatenation layer was subjected to a sigmoid non-linearity activation function and trained using binary cross-entropy loss to get the final corneal mask. Attention gates aided in learning the semantically important features. This technique increases segmentation accuracy for the dataset where tiny RoI features may be lost in cascading convolutions. Furthermore, the model can learn more location-aware features in relation to the classification objective. The corneal mask generated by MS-CNN is used to automatically crop the RoI. The bounding rectangular region around the maximal contour is automatically cropped in the generated mask and used in the classification phase.

### 3.4. Disease Classification

For classifying the RoI cropped images into FK and non-FK classes, transfer learning (with ImageNet [30] pre-trained weights) based on the ResNeXt50 [31] architecture has been employed. ResNeXt50 is modularized based on VGG [32] and ResNet [33]. The multiple paths of ResNeXt50 share the same topology, and it has substantially fewer parameters than VGG. The coarse localization map (before *AdaptiveAvgPool2d*) of the last convolution layer represents the essential features in the input image to detect FK. Grad-CAM calculates attention scores based on gradients determined for the FK output. The attention scores are then normalized and resized to the size of the original image.

## 4. Experimental Results

A detailed discussion on the experimental evaluation of the proposed methodology and the observations are presented in this section. For the implementation and training of the proposed approach, we utilized Python 3.8.8 with the Torch 1.8.0, Keras 2.4.3, and TensorFlow 2.2.0 as backend, running on Ubuntu OS with four NVIDIA Tesla V100-DGXS 32GB GPUs with CUDA v11.2. Based on the normal distribution of available images, the images are resized to (width=384×height=256) dimensions. As per the available system configuration, the batch size is set to 32 images. The model is trained for a maximum of 30 epochs, overall ten folds of the hold-out validation [34].

We used several standard metrics for validating the proposed approach. Dice similarity coefficient (DSC) or $F_{1}$ (with configuration parameter β=1) score and accuracy (refer Equation (1)) are used as primary metrics for validating the output over *C* classes. Dice coefficient is a weighted harmonic mean of positive predictive value (PPV) and true positive rate (TPR), and it seeks to strike a balance between the two (see Equation (2)). Both true/false positives (TP and FP) and true/false negatives (TN and FN) are accounted for in the dice coefficient/F1 measure. Thus, it is more informative than the conventional accuracy score. The positive and negative predictive values(NPV) are computed using Equation (3). True positive and negative rates are computed as per Equation (4).

(1) $$\mathrm{Accuracy}=\frac{1}{C}\;\sum_{c=1}^{C}\frac{{\mathrm{TP}}_{c}+{\mathrm{TN}}_{c}}{{\mathrm{TP}}_{c}+{\mathrm{TN}}_{c}+{\mathrm{FP}}_{c}+{\mathrm{FN}}_{c}}\;$$

(2) $$F_{\beta =1}=\left(1+\beta^{2}\right)\cdot \frac{\mathrm{PPV}\cdot \mathrm{TPR}}{(\beta^{2}\cdot \mathrm{PPV})+\mathrm{TPR}}$$

(3) $$\mathrm{PPV}=\frac{1}{C}\;\sum_{c=1}^{C}\frac{{\mathrm{TP}}_{c}}{{\mathrm{TP}}_{c}+{\mathrm{FP}}_{c}};\;\mathrm{NPV}=\frac{1}{C}\;\sum_{c=1}^{C}\frac{{\mathrm{TN}}_{c}}{{\mathrm{TN}}_{c}+{\mathrm{FN}}_{c}}$$

(4) $$\mathrm{TPR}=\frac{1}{C}\;\sum_{c=1}^{C}\frac{{\mathrm{TP}}_{c}}{{\mathrm{TP}}_{c}+{\mathrm{FN}}_{c}};\;\mathrm{TNR}=\frac{1}{C}\;\sum_{c=1}^{C}\frac{{\mathrm{TN}}_{c}}{{\mathrm{TN}}_{c}+{\mathrm{FP}}_{c}}$$

**Figure 3:**
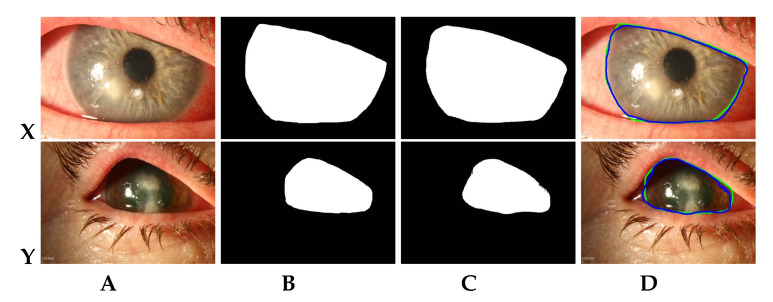
Sample of fully-automatic segmentation results by MS-CNN on diffuse white light images. (**A**) Original images (source: [12]); (**B**) Actual masks obtained for images in (**A**); (**C**) Predicted masks for images in (**A**) using MS-CNN; (**D**) Contour plots of actual (green) and predicted (blue) masks on original images.

The performance of the proposed MS-CNN model is observed using seven-fold cross-validation on all 133 diffuse white light images provided by Loo et al. [12]. We also ensured that the training and testing sets are independent. Table 1 lists the average dice similarity coefficient (DSC) values of MS-CNN and state-of-the-art corneal limbus segmentation techniques. As is evident from Table 1, the proposed MS-CNN outperformed the state-of-the-art model, SLIT-Net [12], by a margin of 1.42%. Furthermore, MS-CNN requires only 5.67 million training parameters compared to 44.62 million for SLIT-Net, which is a 7× reduction. As a result, the proposed MS-CNN is capable of faster training and inference while still enabling more accurate learning of the RoI even with variable sized input images. Figure 3 shows a few samples of actual and predicted corneal region segments for the second test fold. It can be observed that the actual and segmented corneal limbus are in good agreement (see Figure 3D).

**Figure 4:**
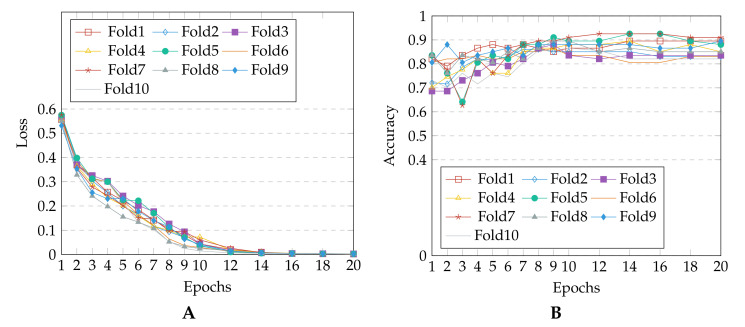
Observations w.r.t each of the 10 folds: (**A**) Loss vs. number of epochs (**B**) Accuracy vs. number of epochs.

**Figure 5:**
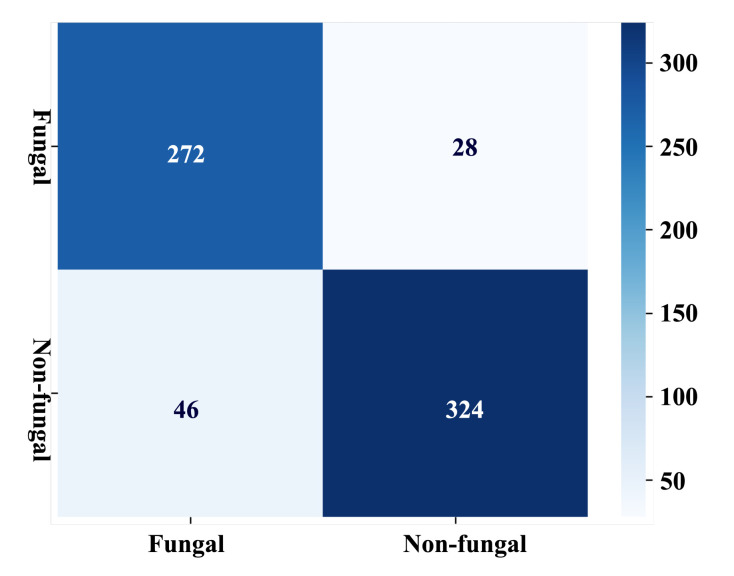
Confusion matrix obtained with ten-fold CV using our model.

**Figure 6:**
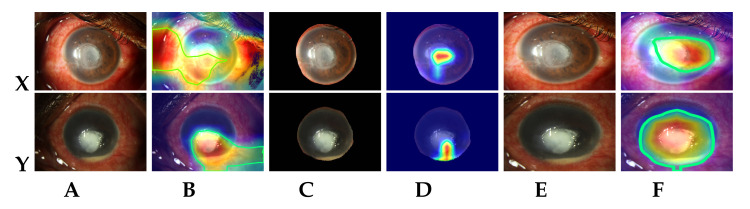
Sample Grad-CAM visualizations generated by the proposed model for correctly identified FK images. (**A**) Original keratitis images (source: [12]); (**B**) Grad-CAM visualizations for (**A**) images; (**C**) Automatic segmented corneal images in (**A**) using MS-CNN; (**D**) Grad-CAM visualizations for (**C**) images; (**E**) Automatic RoI cropped images in (**A**) using MS-CNN; (**F**) Grad-CAM visualizations for (**E**) images.

The FK detection training loss for each fold and accuracy obtained with each test fold are plotted in Figure 4. It can be seen that the loss converges after 12 epochs for all the folds. The accuracy stabilizes after a few initial variations, and the weights with which the model achieved highest precision are saved for each fold. Table 2 presents the details of prediction performance in terms of standard metrics. The confusion matrix obtained for all the ten test folds is shown in Figure 5. The dominant features learned by the model to detect FK are visualized using gradient-weighted class activation mapping (Grad-CAM) [25]. Figure 6 shows the Grad-CAM visualization for the correctly diagnosed patients having FK. The maximal contour (shown using green marking in Figure 6) is drawn using the heatmap mask produced with a threshold value of 100.

## 5. Discussion

During the experiments, we observed that the losses and accuracy between the folds are almost the same (refer Figure 4), proving that the proposed model (with cropping) is generalizable for keratitis diagnosis, despite being trained on varied dimension images. This can be attributed to the effectiveness of the data augmentation and proposed RoI cropping process, which enabled strong focus on the FK lesions while avoiding any over-fitting. To understand the role and importance of RoI cropping process in the prediction pipeline, we also experimented with model training using the original (non-cropped) images. The results revealed a higher variation in the between-fold mean and confidence interval values, when original images (without cropping) were used. This may be attributed to the fact that the model focuses on non-corneal areas (mainly conjunctiva region) for most of the identified FK images. However with cropping, the model is able to distinctly focus and learn the features from dominant lesions like epithelial defect, immune ring, satellite lesions, feathery margins and deep stromal infiltration for detecting FK (refer Figure 6).

An ablation study was carried out for determining the contributions of various individual modules in the proposed FK detection approach. The results obtained for the first fold with the experimented methods have been summarized in Table 3. For obtaining the direct segmented corneal images, we used an approach similar to that proposed by Hung et al. [23]—the pixels within the generated corneal mask were retained, while the remaining pixels in the original input image were set to zero. The segmented masks thus generated had noisy boundaries, clipped cornea regions and many black (zero valued) pixels, resulting in unfocused images. Scaling these images during training data preparation further deteriorated the images, making it difficult for the model to learn the required features. This is evident from the visualized heatmaps (see Figure 6D). When the direct segmented corneal images were used, the model failed to identify the required corneal lesions. In the proposed approach, RoI was initially cropped by using the bounding box around the generated corneal mask contour. Therefore, it is evident that scaling had minimal impact on the model’s performance. The ablation study also revealed that the model’s predictive performance degraded significantly with the absence of transfer learning (i.e., pre-trained weight initialization). Due to random initialization of network parameters when transfer learning is not used, inconsistent results were observed during each training run. In order to attain convergence and reliable results, the network must be trained over a larger number of epochs.

Furthermore, the proposed approach correctly identified most cases of fungal and non-fungal (viral & bacterial) keratitis (refer confusion matrix shown in Figure 5). While false positive instances were noted in cases of acanthamoeba keratitis images, false negative instances appeared to be due to a lack of significant infiltration in the FK images. The latter could be attributed to images of patients who might have been in early stages of the disease or are undergoing medical or surgical treatment, thereby altering the morphology of the infectious infiltrate. In order to address the false negative performance, the poor quality images were removed, and more FK images were included than class-wise non-FK images. We believe that this may have improved our model’s performance. Furthermore, it is to be noted that public-domain images often exhibit distinctive FK features. The data used in our study included a greater prevalence of viral keratitis, whose clinical characteristics are known to be manifestly distinct from those of FK. These differences may have aided the model’s performance as well. However, in order to achieve a high degree of precision, variability of the datasets is essential. Therefore, we plan to address this limitation, by generating datasets with additional acanthamoeba, bacterial keratitis images, and also high-quality images, in our future work. Our model detects FK primarily through identification of morphological changes in the corneal area, but the challenge of identifying a non-infectious corneal infiltrate, or a species-specific form of keratitis is yet to be addressed. Not just micro-organisms, but different species within a group can cause varied ocular signs based on the presence or absence of polymicrobial association and/or periocular conditions. For example, candidal keratitis can cause a collar-stud like morphology as opposed to Fusarium keratitis that causes feathery branch–like extensions or a ring-shaped infiltrate. Viral keratitis in particular is very distinct in its corneal lesions. For example, epithelial keratitis due to Herpes Simplex Virus can present as a dendritic ulcer or a geographical ulcer. It can also present as interstitial keratitis, necrotizing keratitis and disciform keratitis based on the corneal layer primarily involved. This makes it difficult to identify the specific causative organism for the MK. These issues will be included in the future scope of our study.

## 6. Conclusions

Early diagnosis of FK is essential for clinical decision-making and can potentially eliminate vision impairment. Existing manual screening approaches and corneal scraping for microbiological culture-senstivity tests are cumbersome and time-consuming. In this paper, we presented a multi-scale CNN model for automatic segmentation of corneal region combined with ResNeXt neural model for automated FK diagnosis. The Grad-CAM learnt features are visualized to illustrate the interpretability of the proposed pipeline, thereby instilling trust in intelligent healthcare systems. Experimental results show that the proposed MS-CNN trained for segmentation of corneal region achieved superior performance for SLIT-Net dataset, underscoring its effectiveness against state-of-the-art methods. In future, we aim to collate more acanthamoeba keratitis images to further improve the performance of the model and reduce the number of false positives. We also intend to compare the predictive performance of MS-CNN for the segmentation of other corneal lesions like corneal edema border, ulcer border, extent of stromal infiltrate and height of hypopyon.

## Figures and Tables

**Table 1:** Summary of average DSC of the proposed MS-CNN and state-of-the-art corneal limbus segmentation methods, using diffuse white light images (Loo et al. [12]).

Method	DSC (%)	Confidence Interval (*with 0.05 Significance Level*)	Training Parameters (*in Millions*)
U-Net [12]	91	74–100%	34.51 [28]
U${}^{2}$ Net [24]	95.10	93.54–96.66%	44.01
SLIT-Net [12]	95	93–97%	44.62
MS-CNN	96.42	95.65–97.19%	5.67

**Table 2:** Performance evaluation of proposed approach with standard metrics.

Metric	Mean Value	Confidence Interval (*with 0.05 Significance Level*)
Accuracy	88.96%	87.43–90.48%
Sensitivity/Recall/TPR	90.67%	87.95–93.39%
Specificity/TNR	87.57%	85.45–89.69%
Precision/PPV	85.65%	83.59–87.75%
Negative predictive values/NPV	92.18%	90.01–94.33%
F1/Dice coefficient score/DSC	88.01%	86.32–89.70%

**Table 3:** Results of ablation study conducted on the proposed model.

Model	Accuracy (%) ^*d*^	F1 Score (%) ^*d*^
Proposed approach (Section 3.2 + Section 3.3 + Section 3.4)	89.55	89.23
Original images (Section 3.4)	87.88^−*θ*^	87.59^−*θ*^
Segmented corneal images (Section 3.4)	84.62^−*θ*^	84.62^−*θ*^
RoI cropped images (Section 3.2 + Section 3.3)	76.12^−*η*^	76.12^−*η*^

^*d*^ Proposed RoI cropping process is denoted by *θ*. The transfer learning is represented using *η*. Exclusion of a technique is indicated using −.

## Data Availability

Public domain data was used for the experiments conducted as part of the study.
